# Effects of Organizational Cynicism and Socialization on Nurses' Job Burnout: A Career-Stage-Based Analysis

**DOI:** 10.1155/jonm/8883236

**Published:** 2025-09-28

**Authors:** Hee Jin Kim, Hae Jeong An, Soo-hyun Nam, Jungmin Lee

**Affiliations:** ^1^Graduate School of Nursing Sciences, Hallym University, Chuncheon, Gangwon-do, Republic of Korea; ^2^School of Nursing, Ewha Womans University, Seoul, Republic of Korea; ^3^School of Nursing Science, Gyeongkuk National University, 1375 Gyeongdong-ro, Andong 36729, Republic of Korea; ^4^School of Nursing, Hallym University, Chuncheon, Gangwon-do, Republic of Korea

**Keywords:** nurses, organizational cynicism, organizational socialization, professional burnout, workplace stress

## Abstract

This study examined the influence of organizational cynicism and socialization on job burnout among nurses at different career stages, aiming to provide foundational data for efficient workforce management. Data were collected from 271 clinical nurses at H University Hospital September 10–25, 2023. Participants completed a survey consisting of 70 questions covering general characteristics, job burnout, organizational cynicism, and organizational socialization. We conducted analyses using descriptive statistics, *t*-tests, analyses of variance, and correlation and hierarchical regression. Job burnout varied significantly based on gender, while organizational cynicism and socialization showed significant differences according to age and career status. Organizational cynicism was higher among nurses with 13–25 years of experience compared with those with four months to one year of experience. Organizational socialization scores were highest among nurses with 4 months to 1 year of experience. Moreover, job burnout and organizational cynicism were positively correlated, while job burnout and organizational socialization were negatively correlated. Organizational cynicism and socialization were also negatively correlated. Importantly, organizational socialization was identified as a key factor explaining job burnout. We confirmed that job burnout is correlated with organizational cynicism and socialization, indicating an interdependent relationship. For efficient workforce management, programs should be implemented that help reduce or prevent job burnout, build trust, and foster a positive, supportive organizational culture. This underscores the importance of continuous organizational socialization programs for new nurses and resocialization programs for experienced ones.

## 1. Introduction

The global nursing workforce faces growing challenges, such as increasing job stress, high turnover, and retention difficulties, especially in tertiary care settings [1, 2]. In South Korea, these challenges are amplified by rigid hierarchical systems, high patient-to-nurse ratios, and rapid changes in healthcare delivery [3, 4]. Such conditions contribute to psychological burdens such as job burnout and organizational cynicism while also hindering effective organizational socialization. Understanding the interplay among these factors is crucial for sustaining a healthy work environment and ensuring high-quality patient care.

The nursing workforce in hospitals comprises a diverse range of ages and experience levels, reflecting a broad spectrum of professional values and behaviors shaped by different stages of socialization. A 2018 survey indicated that 51.4% of nurses in tertiary hospitals had less than five years of experience, while 17.8% had more than 15 years of experience [5]. Such diversity in experience can produce intergenerational conflicts, as varying belief systems and values lead to misunderstandings and internal strife [6]. Exacerbated by the pursuit of work–life balance, such conflicts contribute to burnout and a deteriorating organizational culture [7, 8]. Burnout is a psychological syndrome that develops in response to prolonged occupational stress and is characterized by emotional exhaustion, depersonalization, and a diminished sense of personal accomplishment [9]. It not only affects individual nurses' well-being but also reduces work efficiency and disrupts staff retention, ultimately affecting nurses' health [10].

In Korea, hospitals are typical hierarchical structures with high patient-to-nurse ratios, particularly in tertiary and general hospitals, exacerbating nurses' already stressful working conditions [11]. Korea's medical environment has undergone rapid systemic and technological changes, requiring nurses to continuously adapt to new demands and expectations [12]. This causes nurses to experience work overload, receive insufficient support, and face increased demands for high-quality care, leading to heightened levels of burnout, particularly among those dealing with ill-mannered or excessively demanding patients [6, 13]. Nurses are highly prone to burnout, with rates approximately twice as high as those of other healthcare professionals, such as social workers and doctors [14]. Burnout can manifest as indifference, cynicism, and psychological detachment, negatively affecting both personal lives and professional performance, leading to increased turnover and diminished quality of care [15–17].

Burnout is linked to organizational cynicism, which is characterized by distrust of and frustration with the organization and can further exacerbate the challenges faced by healthcare institutions [18]. Widespread cynicism can hinder the effectiveness of organizational strategies and produce resistance to change [19, 20]. Managing organizational cynicism is therefore crucial for maintaining a positive work environment and ensuring hospital organizations' continuous development.

Organizational socialization helps nurses adapt to their roles, internalize organizational values, and develop necessary work skills [21]. Socialization not only benefits new nurses but is also vital for experienced nurses who are navigating changes in their work environments or personal lives [22, 23]. Successful socialization can reduce job burnout and turnover rates among experienced nurses, contributing to a more stable, productive workforce [24].

Using a deductive research design and structural equation modeling, Parmar et al. [16] found that organizational cynicism had a significant direct effect on job burnout and that organizational commitment mediated the relationship between burnout and turnover intention. Referring to that theoretical framework, the present study developed a research model to examine the effects of organizational cynicism and socialization on job burnout. Furthermore, by comparing the predictors of job burnout across different career stages, we offer practical guidance for workforce management as well as strategies to enhance organizational adaptation.

Despite an increased focus on burnout's effect on individual nurses and healthcare organizations, few studies have specifically explored how burnout influences organizational cynicism and socialization—particularly across different stages of nurses' careers. Previous studies have mainly treated these constructs in isolation or without considering variations by career stage. Although many studies conceptualize job satisfaction as a precursor to burnout, others have positioned it as a concurrent or contextual factor that shapes how other organizational variables affect burnout outcomes [25, 26].

Job satisfaction and job burnout are closely related affective constructs that can co-occur and mutually influence perceptions of organizational climate [27]. Job satisfaction reflects a broad evaluation of one's work environment, whereas burnout captures a stress-related syndrome arising from prolonged occupational demands. Since satisfaction can shape how nurses perceive and respond to organizational factors such as cynicism and socialization—independent of their level of burnout—prior nursing studies examining burnout in the context of organizational culture or psychosocial climate adjusted for job satisfaction to account for this potential overlap [28, 29]. Following this rationale, we considered job satisfaction as a covariate to better isolate the unique associations among burnout, organizational cynicism, and organizational socialization.

## 2. Methods

### 2.1. Research Design

This study used a cross-sectional, descriptive correlational design to examine the relationship between job burnout, organizational cynicism, and organizational socialization among clinical nurses at various stages of their careers.

### 2.2. Participants

Nurses working at H University Hospital in Gyeonggi Province, South Korea, were randomly sampled for this study. The inclusion criteria were as follows: (1) adults aged 18 years or older, (2) individuals currently working as nurses, and (3) nurses with work experience of 4 months or more. The exclusion criteria were individuals in nursing management positions (head nurses or higher) and those with less than 4 months of work experience. We used G∗Power 3.1.9.4 to calculate the sample size and select the participants. For one-way analysis of variance (ANOVA) with an effect size of 0.25, a significance level of 0.05, a power of 0.95, and five predictors, the required sample size was calculated to be 230. To account for a potential dropout rate of 20%, we distributed questionnaires to 280 nurses. Nurses were informed about the purpose of the study and assured that there would be no penalty for nonparticipation. All participants voluntarily consented to participate in the study. Finally, data from 271 participants were analyzed after excluding nine questionnaires with incomplete or inappropriate responses.

### 2.3. Measurements

#### 2.3.1. General Characteristics

This section of the questionnaire comprised 10 demographic items: gender, age, marital status, highest level of education, clinical experience, current position, experience with department transfers, current department, work type, and satisfaction with work.

#### 2.3.2. Job Burnout

We used the Copenhagen Burnout Inventory, which was developed by Kristensen et al. [30] to measure job burnout in service workers and later adapted for nurses by Ham [31]. The inventory comprises 19 items divided into three subareas: personal, work-related, and client-related burnout. It uses a five-point Likert scale, ranging from 1 (*not at all*) to 5 (*always*), with one item (item 10) being reverse-scored. Scores range from 19 to 95, with higher scores indicating greater burnout. The original instrument [30] had Cronbach's *α* of 0.89; in Ham [31], it was 0.93. In our study, Cronbach's *α* was 0.93.

#### 2.3.3. Organizational Cynicism

To measure organizational cynicism, we used six items developed by Atwater et al. [32] and adapted by Choi [33] to align with the organizational contexts and cultural nuances relevant to social workers. Responses are measured on a five-point Likert scale (1 = not at all to 5 = often), with higher scores indicating greater cynicism. The original instrument [32] had Cronbach's *α* of 0.85; in a previous domestic study [33], it was 0.87. In our study, Cronbach's *α* was 0.74.

#### 2.3.4. Organizational Socialization

Organizational socialization was assessed using a tool developed by Sohn et al. [22]. We used 34 of the original 39 items, excluding those that overlapped with the job burnout section. Responses are measured on a five-point Likert scale (1 = not at all to 5 = very much). The original tool [22] had Cronbach's *α* of 0.97. In our study, Cronbach's *α* was 0.86.

### 2.4. Data Collection

We collected data from September 10 to September 25, 2023. Requests for survey cooperation were sent to the nursing department and the managers of each department. We visited each department to explain the study purpose, questionnaire completion process, and data collection methods, emphasizing participant anonymity and confidentiality. We clarified that participation was voluntary and that participants could withdraw from the study at any time without consequences. Only those who provided written consent after being fully informed about the study participated in the survey. This study adhered to the EQUATOR guidelines for cross-sectional studies.

### 2.5. Data Analysis

We used IBM SPSS version 25.0 for data analysis. Descriptive statistics, including means and standard deviations, were used to analyze the responses in all survey sections. We used independent *t*-tests and one-way ANOVA to examine differences in job burnout, organizational cynicism, and organizational socialization according to participants' general characteristics. Levene's test was used to confirm the homogeneity of variances. Post hoc comparisons were performed using Scheffé's test. Correlations among job burnout, organizational cynicism, and organizational socialization were analyzed using Pearson's correlation coefficient, following standard guidelines [34].

We used hierarchical multiple regression to identify the predictors of job burnout. Three models were constructed sequentially to assess the added explanatory power of general characteristics (Model 1), organizational cynicism (Model 2), and organizational socialization (Model 3) [35].

### 2.6. Ethical Considerations

This study was approved by the Institutional Review Board of H University (approval no.: ANONYMIZED) and conducted in accordance with the Declaration of Helsinki.

## 3. Results

### 3.1. Participants' General Characteristics

Table 1 summarizes the sociodemographic characteristics of the 271 participants. The majority were women (91.1%), with an average age of 30.77 years (SD = 6.72). The age distribution was as follows: 17.7% were aged 22–24 years, 21.4% were 25–27 years, 18.5% were 28–30 years, 21.4% were 31–35 years, and 21.0% were 36–50 years. Regarding religious affiliation, 33.9% of the participants reported observing a religion. Most participants were single (74.9%) and held a bachelor's degree (76.8%), while 11.1% held an associate's degree and 12.2% had attained some higher education.

Average clinical experience was 7.31 years (SD = 6.82). Employment history showed that 13.3% had worked for more than 4 months but less than a year, 18.5% for more than a year but less than 3 years, and 21.8% for more than 3 years but less than 6 years. Most participants were staff nurses (92.6%), while 7.4% were charge nurses. A total of 38.4% reported having departmental transfer experience.

Department distribution was as follows: 16.2% worked in general internal medicine wards, 16.6% in general surgical wards, 17.0% in nursing care integration service wards, 35.4% in specialized units (e.g., ICUs and operating rooms), and 14.8% in outpatient/testing departments. Regarding work patterns, 22.1% were full-time employees, while 77.9% worked rotating shifts. The average job satisfaction score was 3.05 (SD = 0.78), with 54.2% being neutral, 25.5% satisfied, and 18.9% dissatisfied.

### 3.2. Levels of Job Burnout, Organizational Cynicism, and Organizational Socialization

Supporting Table 1 presents the correlation coefficients between job burnout, organizational cynicism, and organizational socialization stratified by participants' career stages (*N* = 271). Pearson's correlation coefficients (*r*) and associated *p* values are reported for five career stage groups: < 1 year, 1-2 years, 3–5 years, 6–12 years, and 13–24 years.

The average job burnout score, measured on a five-point scale, was 3.44 ± 0.66. Among the components, personal burnout was the highest at 3.93 ± 0.70, followed by work-related burnout at 3.27 ± 0.73 and client-related burnout at 3.18 ± 0.77. Organizational cynicism averaged 2.65 ± 0.60 on a five-point scale. For organizational socialization, the average score was 3.07 ± 0.38 on a five-point scale.

### 3.3. Correlation Between Job Burnout, Organizational Cynicism, and Organizational Socialization by Career Duration

Table 2 shows the correlations between job burnout, organizational cynicism, and career duration. Several significant relationships were identified.

A notable finding was the significant positive correlation between job burnout and organizational cynicism for nurses with 3–6 years of experience (*r* = 0.505, *p* < 0.001), indicating that increased burnout at this career stage is linked to higher cynicism. The correlation was the lowest for those with 1–3 years of experience (*r* = 0.288, *p* < 0.042). We found no significant correlation between these variables for nurses with more than 6 years of experience.

Significant correlations were also found between job burnout and organizational socialization across all career stages. The strongest negative correlation was for those with 1–3 years of experience (*r* = −0.649, *p* < 0.001), suggesting that higher burnout correlated with lower socialization in this group. The weakest negative correlation was for nurses with 4–12 months of experience (*r* = −0.381, *p*=0.022).

Similarly, we found significant correlations between organizational cynicism and socialization. The strongest negative correlation was observed in the 1–3 years group (*r* = −0.706, *p* < 0.001), suggesting that higher cynicism is associated with lower socialization at earlier career stages. The weakest correlation was for those with 13–25 years of experience (*r* = −0.409, *p*=0.001), suggesting that this relationship weakens as nurses gain experience.

### 3.4. Hierarchical Regression

Table 3 presents the hierarchical regression results, assessing the effects of participants' general characteristics, organizational cynicism, and organizational socialization on job burnout. Model 1 included general characteristic categories identified as significant predictors of job burnout in previous studies—namely, gender, age, marital status [36], education [37], and satisfaction [38]. Nominal and sequence variables were coded as dummy variables. Model 2 included organizational cynicism, and Model 3 included organizational socialization. The tolerance levels of the regression model ranged from 0.440 to 0.957, and the variance inflation factor ranged from 1.045 to 2.271, indicating no multicollinearity issues and validating the use of hierarchical regression.

Model 1 explained 28% of the variance in job burnout (*F* = 12.681, *p* < 0.001), with gender (*ß* = 0.23) and satisfaction (*ß* = −0.28 to 0.26) emerging as significant predictors. Model 2 explained 30% of the variance in job burnout (*F* = 12.810, *p* < 0.001), representing a 2% increase in explanatory power compared with Model 1. Model 3 explained 41% of the variance in job burnout (*F* = 17.722, *p* < 0.001), representing an 11% increase over Model 2. In Model 3, organizational cynicism no longer significantly affected job burnout, whereas organizational socialization had a significant negative effect.

## 4. Discussion

This study found that job burnout was relatively high among participants, with an average score of 3.44 out of 5. This is higher than in previous studies, such as Nam and Noh [39] with 3.04, Jeong and Choi [40] with 3.32, and Lee [41] with 3.03. This elevated burnout could be attributable to differences in work environments and support systems. For consistency, all comparative studies cited here [39–43] used the same burnout measurement tool (i.e., the Copenhagen Burnout Inventory) and employed identical Likert-type scoring methods (1–5 scales), allowing for valid comparisons. Previous studies of nurses in small- (100 beds or fewer) and medium-sized (100–300 beds) hospitals reported lower burnout levels (e.g., 3.19 in [42]). Conversely, study participants from upper-level hospitals such as the sample institution (H University Hospital), which are tertiary care hospitals with over 500 beds, experienced higher levels of burnout, likely because of the severity of the managed cases. Prior studies of upper-level hospital nurses have similarly reported higher burnout rates (e.g., 3.21 in [39] and 3.10 in [43]). Given the link between burnout and negative emotions [44, 45], additional research is needed to develop effective management strategies.

Participants exhibited moderate organizational cynicism, averaging 2.65 out of 5, which is slightly lower than the 2.72 reported by Kim [46] for outpatient nurses in upper-level hospitals. Higher levels were noted in studies of healthcare workers during the COVID-19 pandemic, such as Kim et al. [47] (3.06) and Jang [48] (3.18), likely due to pandemic conditions and variations in measurement tools. Comparisons across professions also show moderate cynicism levels, with cynicism often manifesting as anger and stress, negatively affecting work performance [49]. Regarding organizational socialization, participants in this study scored an average of 3.55, which was slightly higher than the 3.48 in Kim et al. [47] and 3.14 in Jung and Park [50]. These score variations may be attributable to organizational culture, adaptability, and job roles. Effective socialization is linked to positive outcomes, whereas poor socialization can increase burnout and cynicism [51].

Job burnout and organizational cynicism exhibited the most significant positive correlations among the variables. The highest positive correlation between job burnout and organizational cynicism was observed among participants with 3–6 years of experience, and the lowest was among those with 1–3 years of experience. The stronger correlation in the former suggests that as nurses enhance their clinical judgment and coping skills, they experience higher job burnout, which in turn increases organizational cynicism. We used Fisher transformation to examine whether the correlation between job burnout and organizational cynicism differed significantly by career stage. The results showed a statistically significant difference between early-career (1–3 years) and midcareer (3–6 years) nurses (*z* = 2.14, *p* < 0.05), indicating that the relationship strengthens with experience.

In Supporting Table 1, sample sizes vary by career stage: less than 1 year (*n* = 36), 1–2 years (*n* = 50), 3–5 years (*n* = 59), 6–12 years (*n* = 66), and 13–24 years (*n* = 60). While strong correlations between job burnout and organizational cynicism were evident in the 3–5 years group (*r* = 0.505, *p* < 0.001), this relationship became nonsignificant in the 6–12 and 13–24 years groups (*r* = 0.205 and 0.191, respectively). This trend may reflect a point of inflection where mid-to-late-career nurses develop coping mechanisms, career detachment, or recalibrated expectations, potentially buffering against the burnout–cynicism link. However, the reduction in correlation strength might also be partially attributed to smaller group sizes in later career stages, limiting statistical power. Including these sample sizes provides a critical context for interpreting nonsignificant findings.

Previous studies, including those of Kim et al. [47] and others using different tools [32, 52, 53], have also found a positive correlation between job burnout and organizational cynicism. This indicates that increased levels of job burnout are associated with higher levels of organizational cynicism. These findings also suggest that targeted interventions focusing on stress management and professional development, especially for nurses with 3–6 years of experience, are crucial for mitigating the effects of job burnout on organizational cynicism.

Organizational cynicism and socialization exhibited the most significant negative correlations. The strongest negative correlation was found among participants with 1–3 years of experience, whereas the weakest was found among those with 13–25 years of experience. Increased cynicism can hinder socialization efforts, potentially affecting nurses' attitudes and behaviors within the organization, depending on their career stages. These findings highlight the need for further research on the relationship between organizational cynicism and socialization—particularly for nurses with 1–3 years of experience—to develop evidence-based strategies nursing managers can use to enhance socialization efforts and mitigate the negative effects of cynicism.

In this study, the correlation between job burnout and organizational socialization was negative. The strongest negative correlation was observed among participants with 1–3 years of experience, whereas the weakest was among those with 4–12 months of experience. This aligns with previous studies [49] that also reported a negative correlation between job burnout and organizational socialization. The negative correlation suggests that higher levels of job burnout are associated with lower levels of organizational socialization. This underscores the importance of exploring job burnout's effect on organizational socialization in early-career nurses and providing a foundation for nursing managers to develop targeted interventions that enhance socialization while addressing burnout.

Hierarchical regression confirmed that organizational socialization significantly contributes to job burnout. In Model 3, adding organizational socialization increased explanatory power by 11%, making organizational cynicism insignificant while highlighting organizational socialization's significant negative effect on job burnout. This suggests that organizational socialization is a key factor explaining job burnout, and enhancing it could help reduce burnout among nurses. Organizational socialization encompasses organizational values, supportive interpersonal relationships, and professional identities [54]. Jung [55] found that organizational atmosphere significantly affected organizational socialization, suggesting that fostering a supportive group atmosphere and professional identity among nurses could improve socialization and reduce burnout. Research on the relationship between organizational socialization and job burnout is sparse. We propose that organizational socialization is a determining factor in job burnout, underscoring the need for additional research in this area.

We selected hierarchical regression to examine the incremental explanatory power of key predictors over and above basic demographic variables. Model 1 only included general characteristics, establishing a baseline for explaining job burnout. Model 2 added organizational cynicism to assess its unique contribution while controlling for demographic factors. In Model 3, organizational socialization was entered to determine whether it accounted for additional variance beyond demographics and cynicism. This stepwise inclusion approach allowed us to evaluate not only the direct effects of each construct but also the potential suppression or attenuation of effects when variables were considered simultaneously. The observed reduction of organizational cynicism's significance in Model 3, alongside the substantial increase in *R*^2^ (from 0.30 to 0.41), suggests that socialization may function as a protective factor that offsets cynicism's detrimental effects on burnout. This interpretation aligns with the methodological guidance of hierarchical modeling, where changes in explained variance and shifts in coefficient significance across models provide insight into the interplay and relative strength of predictors [35]. By explicitly structuring the regression in this way, we could capture the layered influence of individual and organizational factors, thereby enhancing the theoretical and practical implications of our findings. This approach also strengthens the evidential basis for stage-specific workforce strategies, highlighting organizational socialization as a critical leverage point for reducing burnout.

Our findings underscore the need for managers to adopt stage-specific approaches to workforce retention. In particular, midcareer nurses experiencing increasing organizational cynicism require targeted leadership support, peer recognition programs, and decision-making involvement to restore trust and reduce disengagement. The implications for nurses are substantial. Persistent burnout and increasing cynicism not only deteriorate nurses' psychological well-being—leading to emotional exhaustion, low morale, and reduced job engagement—but also compromise clinical performance, teamwork, and patient safety. In the long term, these effects may result in increased absenteeism, higher turnover rates, and reduced career longevity among skilled nurses. Therefore, the management of burnout and promotion of organizational socialization are not merely administrative concerns but essential strategies for protecting the functional and emotional health of the nursing workforce. Nurse managers must also reinforce ongoing organizational socialization, not just during onboarding but as a longitudinal strategy integrated into professional development and performance management systems.

### 4.1. Theoretical and Practical Implications

Theoretically, this study highlights the need to conceptualize job burnout, organizational cynicism, and socialization as interrelated psychosocial constructs that evolve across career stages. We add depth to prior research by focusing on how the meaning and effect of these factors shift over time in a nurse's career. Notably, the role of organizational socialization extends beyond early onboarding and plays an ongoing role in shaping nurses' long-term adaptation and psychological well-being.

Practically, our results emphasize the need for differentiated interventions. For early-career nurses, structured onboarding and mentorship may reduce burnout and support socialization. For midcareer nurses, professional development, leadership opportunities, and stress-management programs may reduce cynicism and reengage motivation. Healthcare institutions should consider integrating burnout prevention strategies with socialization initiatives to foster both individual resilience and a healthy organizational culture.

### 4.2. Limitations

This study has several limitations. First, the cross-sectional design limited its ability to establish causality. Although we observed significant correlations, we could not definitively determine whether one variable influenced changes in another. Longitudinal research is needed to better understand the causal relationships among these variables. Second, our findings were based on a specific group of nurses from a single hospital, potentially limiting their generalizability to other hospitals or regions. Nurses' experiences and work environments can vary significantly across different healthcare institutions and geographical regions. Finally, the tools we used were adapted to the sociocultural context of Korea and might not be equally applicable in other cultural settings. Future research should therefore conduct cross-cultural validation studies to evaluate the reliability and applicability of these instruments in diverse cultural and work environments. Future studies could also develop or incorporate culturally neutral instruments to enhance the generalizability of their findings. Acknowledging these limitations is crucial for accurately interpreting our findings and guiding future research in this area. Addressing these limitations can help enhance our understanding of the complex dynamics of the nursing work environment.

Overall, future studies should use longitudinal or mixed-method designs to explore the causal relationships between burnout, cynicism, and socialization over time. In particular, qualitative research could help illuminate how nurses experience socialization and burnout in different institutional cultures. Additionally, future work should consider testing and refining interventions—such as structured socialization programs, peer-support groups, or leadership training—to evaluate their effectiveness for reducing burnout and promoting professional identity across various career stages.

## 5. Conclusion

This study found that job burnout levels were higher among nurses in advanced general hospitals than in smaller hospitals, likely due to the increased workload and stress associated with managing more severe patient conditions. We observed significant differences in organizational cynicism and socialization, particularly based on career length, with midcareer nurses showing higher levels of cynicism, which could negatively affect hospital culture.

These findings suggest that early-career organizational socialization may diminish over time, highlighting the need for tailored socialization strategies. For nurses with 1–3 years of clinical experience, enhanced onboarding or mentorship programs could strengthen early socialization and foster a sense of belonging, thereby reducing burnout and cynicism. For those with 3–6 years of experience, stress management and professional development initiatives could help address the challenges associated with their increased responsibilities. Hospitals should proactively design career-stage-specific strategies that not only address current stressors but also anticipate and prevent burnout-related disengagement over time. This includes building frameworks that emphasize organizational values and promote supportive relationships and professional identity through team-building activities and peer-support programs.

Importantly, our findings underscore the strategic value of investing in long-term organizational socialization——not merely as a one-time onboarding process but as a dynamic, ongoing mechanism for building engagement, resilience, and retention across all stages of a nurse's career. By embedding socialization efforts within broader institutional policies—such as leadership development, participatory governance, and mental health support—organizations can shift from reactive burnout management to proactive workforce sustainability planning.

Future workforce strategies in healthcare should prioritize not only clinical skill development but also psychosocial integration, team cohesion, and organizational adaptability. Our study suggests that sustained investment in socialization processes can potentially transform organizational culture by empowering nurses as resilient, engaged professionals equipped to navigate evolving healthcare demands.

## Figures and Tables

**Table 1:** General characteristics of the participants (*N* = 271).

Characteristics	Categories	*n* (%)	*M* ± SD
Gender	Men	24 (8.9)	
	Women	247 (91.1)	

Age (years)	22–24	48 (17.7)	30.7 ± 6.7
	25–27	58 (21.4)	
	28–30	50 (18.5)	
	31–35	58 (21.4)	
	36–50	57 (21.0)	

Religious beliefs	Yes	92 (33.9)	
	No	179 (66.1)	

Marital status	Single	203 (74.9)	
	Married	68 (25.1)	

Educational background	Associate's degree	90 (11.1)	
	Bachelor's degree	208 (76.8)	
	Graduate degree or higher	33 (12.2)	

Clinical experience (years)	< 1	36 (13.3)	7.31 ± 6.82
	1–2	50 (18.5)	
	3–5	59 (21.8)	
	6–12	66 (24.4)	
	13–24	60 (22.1)	

Current position	Staff nurse	251 (92.6)	
	Charge nurse	20 (7.4)	

Department transfer experience	Yes	104 (74.9)	
	No	167 (25.1)	

Current department	General internal medicine wards	44 (16.2)	
	General surgical wards	45 (16.6)	
	Nursing care integration service wards	46 (17.0)	
	Specialized units^∗^	96 (35.4)	
	Outpatient/testing departments^∗∗^	40 (14.8)	

Work patterns	Full time	60 (22.1)	
	Rotating shifts	211 (77.9)	

Job satisfaction	Completely dissatisfied	11 (4.1)	3.05 ± 0.78
	Dissatisfied	40 (14.8)	
	Neutral	147 (54.2)	
	Satisfied	69 (25.5)	
	Very satisfied	4 (1.5)	

^∗^Intensive care unit, emergency room, operating room, and dialysis unit.

^∗∗^Endoscopy room, full-time staff.

**Table 2:** Job burnout, organizational cynicism, and organizational socialization (*N* = 271).

Category	Average score		
	*M* ± SD	Min	Max
Job burnout	3.44 ± 0.66	1.63	5.00
Personal	3.93 ± 0.70	1.83	5.00
Work related	3.27 ± 0.73	1.17	5.00
Client related	3.18 ± 0.77	1.29	5.00
Organizational cynicism	2.65 ± 0.60	1.00	4.33
Organizational socialization	3.07 ± 0.38	1.79	4.03
Personal characteristics	3.58 ± 0.54	1.38	4.88
Occupational identity	3.37 ± 0.70	1.00	5.00
Collective characteristics	3.06 ± 0.53	1.13	4.50
Job performance	3.03 ± 0.78	1.00	5.00
Job satisfaction	2.82 ± 0.62	1.00	4.80
Organizational commitment	2.41 ± 0.67	1.00	4.20

**Table 3:** Hierarchical regression analysis for job burnout.

Variables	Model 1	Model 2	Model 3
	*β* (*p*)	*β* (*p*)	*β* (*p*)
Age	0.04 (0.576)	0.01 (0.831)	0.03 (0.603)
Gender (ref = men)			
Women	0.23 (< 0.001)	0.22 (< 0.001)	0.19 (< 0.001)
Marital status (ref = single)			
Married	−0.15 (0.023)	−0.14 (< 0.030)	−0.11 (0.056)
Educational background (ref = graduate degree or higher)			
Associate's degree	0.02 (0.804)	0.03 (0.664)	0.5 (0.504)
Bachelor's degree	−0.02 (0.791)	−0.02 (0.793)	−0.03 (0.662)
Job satisfaction (ref = neutral)			
Completely dissatisfied	0.26 (< 0.001)	0.25 (< 0.001)	0.14 (0.006)
Dissatisfied	0.20 (< 0.001)	0.16 (0.004)	0.08 (0.105)
Satisfied	−0.28 (< 0.001)	−0.23 (< 0.001)	−0.14 (0.010)
Very satisfied	−0.12 (0.025)	−0.11 (0.034)	−0.10 (0.034)
Organizational cynicism		0.18 (0.002)	−0.04 (0.522)
Organizational socialization			−0.45 (< 0.001)
*F* (*p*)	12.681 (< 0.001)	12.810 (< 0.001)	17.722 (< 0.001)
*R* ^2^	0.30	0.33	0.43
Adjusted *R*2	0.28	0.30	0.41

## Data Availability

The datasets used in this study are available from the corresponding author upon reasonable request.

## Supporting Information

Supporting Table 1: Correlations between variables according to participants' career stages (*N* = 271).
