# Supplementary material for: Effects of Organizational Cynicism and Socialization on Nurses' Job Burnout: A Career-Stage-Based Analysis
Source: J Nurs Manag. 2025 Sep 28;2025:8883236. doi: 10.1155/jonm/8883236 (PMC12497523; doi:10.1155/jonm/8883236)
Supplement: Supporting Information — Additional supporting information can be found online in the Supporting Information section. [file 8883236.f1.docx]

**Supplementary Table 1** Correlations between variables according to participants’ career stages (N = 271).

| **Career (years)** | **<1** | | | **1–2** | | | | **3–5** | | | | **6–12** | | | | | **13–24** | | | |
| --- | --- | --- | --- | --- | --- | --- | --- | --- | --- | --- | --- | --- | --- | --- | --- | --- | --- | --- | --- | --- |
| **Variables** | JB | OC | OS | JB | OC | OS | JB | | OC | OS | JB | | OC | | OS | JB | | OC | OS |  |
|  | r(*p*) | | | r(*p*) | | | | r(*p*) | | | | r(*p*) | | | | | r(*p*) | | | |
| **JB** | 1 |  |  | 1 |  |  | 1 | |  |  | 1 | |  |  | | 1 | |  |  |  |
| **OC** | 0.501^**^  (0.002) | 1 |  | 0.288^*^  (0.042) | 1 |  | 0.505^***^  (<0.001) | | 1 |  | 0.205  (0.098) | | 1 | |  | 0.191  (0.143) | | 1 |  |  |
| **OS** | -0.381^*^  (0.022) | -0.648^**^  (<0.001) | 1 | -0.649^***^  (<0.001) | -0.706^***^  (<0.001) | 1 | -0.605^***^  (<0.001) | | -0.678^***^  (<0.001) | 1 | -0.575^***^  (<0.001) | | -0.580^***^  (<0.001) | | 1 | -0.544^***^  (<0.001) | | -0.409^**^  (0.001) | 1 |  |

^*^p < 0.05, ^**^p < 0.01, ^***^p < 0.001, JB = Job burnout, OC = Organizational cynicism, OS = Organizational socialization
